# Screening for antiradical efficiency of 21 semi-synthetic derivatives of quercetin in a DPPH assay

**DOI:** 10.2478/intox-2013-0003

**Published:** 2013-03

**Authors:** Ivana Milackova, Lucia Kovacikova, Miroslav Veverka, Ján Gallovic, Milan Stefek

**Affiliations:** 1 Institute of Experimental Pharmacology and Toxicology, Slovak Academy of Sciences, 84104 Bratislava, Slovak Republic; 2 EUROFINS BEL/NOVAMANN Ltd, 940 02 Nové Zámky, Slovak Republic

**Keywords:** antioxidant, quercetin derivatives, DPPH assay, kinetics, stoichiometry

## Abstract

The group of 21 novel semi-synthetic derivatives of quercetin was screened for the antiradical efficiency in a DPPH assay. The initial fast absorbance decrease of DPPH, corresponding to the transfer of the most labile H atoms, was followed by a much slower absorbance decline representing the residual antiradical activity of the antioxidant degradation products. Initial velocity of DPPH decolorization determined for the first 75-s interval was used as a marker of the antiradical activity. Application of the kinetic parameter allowed good discrimination between the polyphenolic compounds studied. The most efficient chloronaphthoquinone derivative (compound Ia) was characterized by antiradical activity higher than that of quercetin and comparable with that of trolox. Under the experimental conditions used, one molecule of Ia was found to quench 2.6±0.1 DPPH radicals.

## Introduction

The antioxidant action of flavonoids, the best described biological activity of this group of natural polyphenolic substances, is covered by a number of excellent reviews (Bors *et al.*, 1990; Cao *et al.*, 1997; Pietta, 2000; Rice-Evans, 2001; Nijveldt *et al.*, 2001; Bors & Michel, 2002; Heim *et al.*, 2002; Williams *et al.*, 2004; Amič *et al.*, 2007; Bischoff, 2008; Boots *et al.*, 2008). Flavonoids exert antioxidant effects by different mechanisms as *e.g.* free radical scavenging, hydrogen donating, singlet oxygen quenching, and metal iron chelating. Within the flavonoid family, quercetin (Qc) is the most potent scavenger of reactive oxygen species, including superoxide, peroxyl, alkoxyl and hydroxyl radicals, and reactive nitrogen species like NO· and ONOO· (Pietta, 2000; Butkovič *et al.*, 2004; Amič *et al.*, 2007; Boots *et al.*, 2008). Flavonoids were found also to scavenge efficiently the model free radicals of 2,2-diphenyl-1-picrylhydrazyl (DPPH) (Butkovič *et al.*, 2004).

Quercetin *O*-glycosides, represent one of the most ubiquitous structures of all plant phenolics (Materska 2008). In addition, synthetic acyl derivatives of Qc, including aliphatic acids such as acetic, malonic and 2-hydroxypropionic acid, or aromatic acids, including benzoic, gallic, caffeic and ferulic acid, are frequently used as synthetic alternative to natural glycoside moieties (Harborne ed., 1994). Acylated Qc derivatives constitute useful active principles for cosmetic, dermatopharmaceutical, pharmaceutical or dietetic compositions (Perrier *et al.*, 2001; Golding *et al.*, 2001). The glycosidic structure has a large impact on quercetin bioavailability (Arts *et al.* 2004; Crozier *et al.* 2010, Stefek and Karasu 2011). The biological activity of Qc derivatives, including their antioxidant action, strongly depends on the nature and position of the substituents. It is important to modify selectively the various hydroxyls, which are not equivalent from either the chemical or biofunctional point of view. The general structural requirements for effective radical scavenging and/or the antioxidant potential of flavonoids are summarized in Bors’ criteria (Bors & Michel, 2002; Amič *et al.*, 2007).

In the present paper, 21 novel semi-synthetic derivatives of Qc were screened for antiradical efficiency in a DPPH assay in comparison with the parent Qc and the standard antioxidant trolox. Stoichiometry of the DPPH quenching reaction was determined for the most efficient derivative.

## Materials and methods

### Chemicals

**Figure 1:**
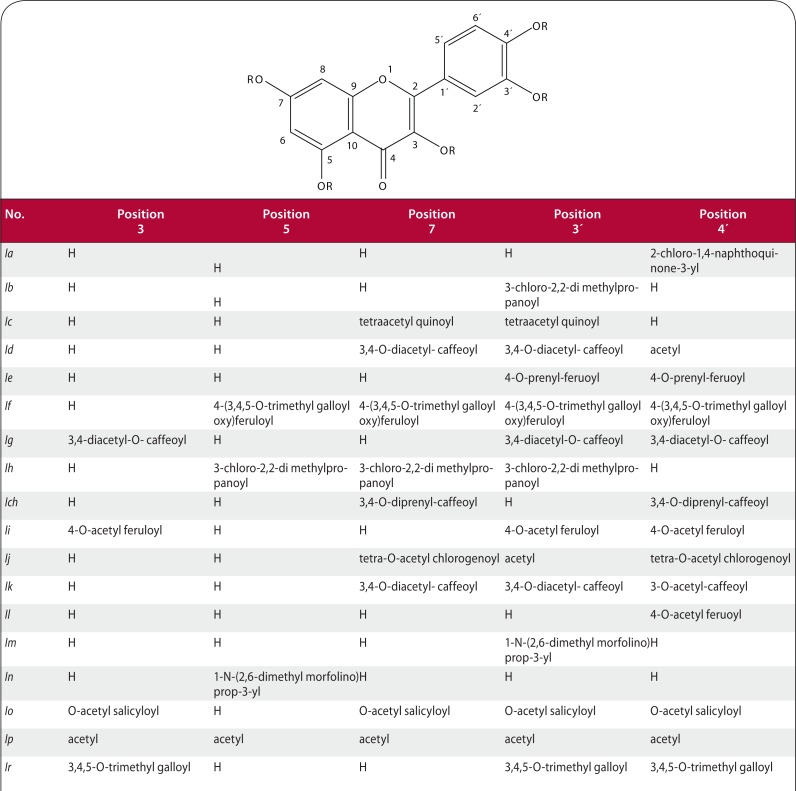
Compounds *Ia*–*Ir*.

**Figure 2:**
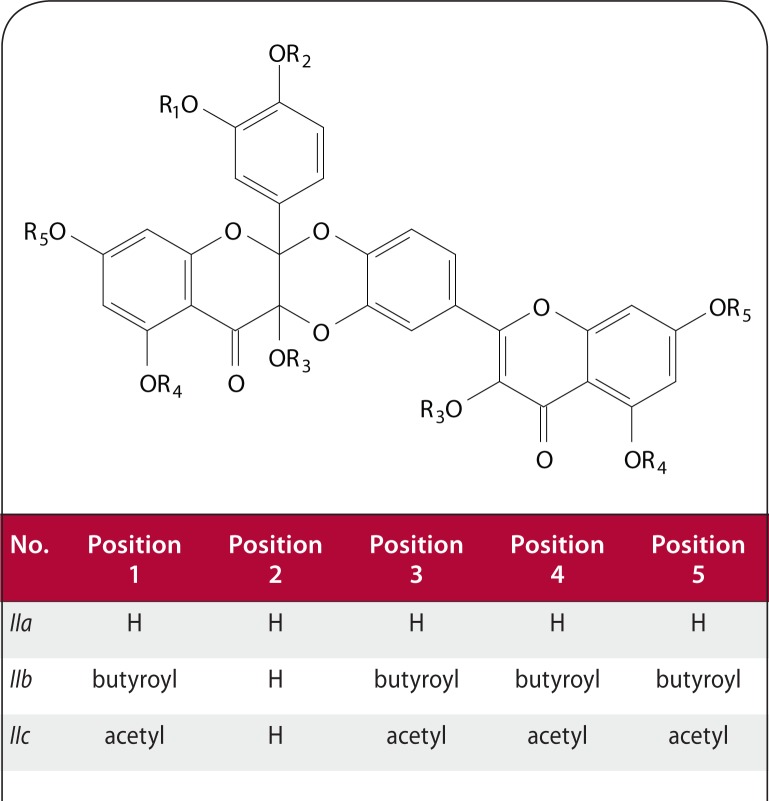
Compounds *IIa*–*IIc*.

Samples of new semi-synthetic derivatives of Qc **Ia–Ir** (Figure 1) were synthesized by reaction of appropriate acyl chloride with Qc or the corresponding protected derivative and then purified by repeated column chromatography of the rich reaction mixture. Qc was oxidized to generate heterodimer **IIa** (Figure 2). Diquercetin was treated with an anhydride to yield corresponding acyl derivatives **IIb–IIc** (Figure 2; Veverka *et al.* 2013).

1,1‘-Diphenyl-2-picrylhydrazyl (DPPH) radical was obtained from Sigma Chemical Co. (St. Louis, MO, USA). Other chemicals were purchased from local commercial sources and were of analytical grade quality.

### DPPH test

To investigate the antiradical activity of the compounds studied, the ethanolic solution of DPPH (50 µM) was incubated in the presence of the given compound tested (50 µM) at laboratory temperature. The absorbance decrease, recorded at λ_max_ = 518 nm, during the first 75-s interval was taken as a marker of the antiradical activity. During the 75-s interval used, an approximately linear decrease of DPPH absorbance was observed, which was considered as a good assessment of the initial velocity of the radical reaction.

The stoichiometry of the radical reaction was determined by spectrophotometric titration of the ethanolic solution of DPPH (50 µM) by increasing concentrations of an antioxidant with the reaction time long enough for completion of the reaction as indicated.

The radical studies were performed at the laboratory temperature.

## Results and discussion

**Figure 3:**
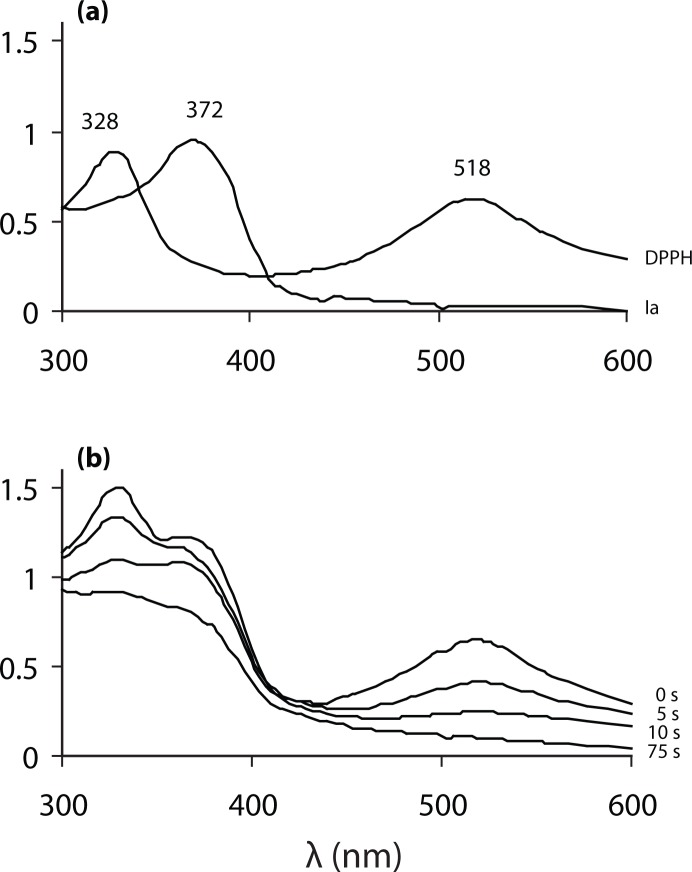
**(a)**. UV-VIS spectra of DPPH (50 µM) and compound **Ia** (50 µM) with characteristic absorbance maxima; **(b)**. Time dependent spectral changes in the mixture of DPPH (50 µM) and compound **Ia** (50 µM).

**Figure 4:**
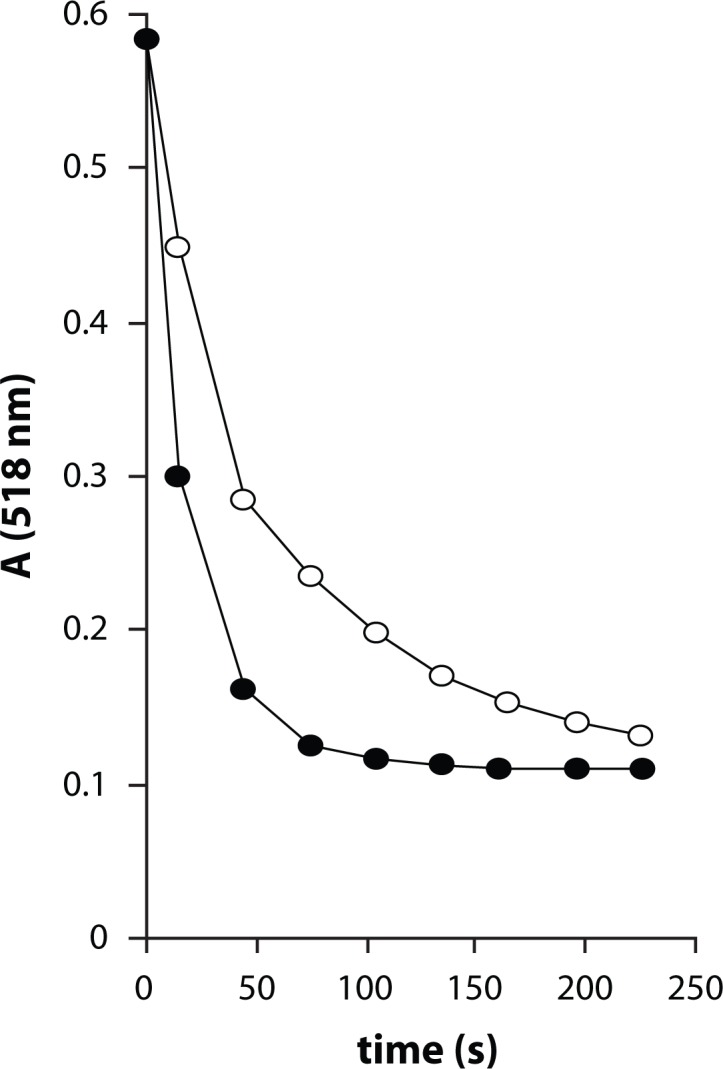
Continual absorbance decrease of ethanolic solution of DPPH radical (50 µmol/l) in the presence of equimolar concentration of tested compounds at λ_max_ = 518 nm. (○)- Qc, (●) - chloronaphthoquinone derivative **Ia**. The curves represent results from two typical experiments.

As a weak hydrogen atom abstractor, DPPH is considered a good kinetic model for peroxyl ROO· radicals (Blois, 1958; Ratty *et al.*, 1988). DPPH assay is routinely used as a primary screening test of antiradical efficacy. Figure 3 shows UV-VIS spectra of DPPH and compound **Ia** with characteristic absorbance maxima and their time-dependent changes during the first 75 sec after mixing the reactants. The time-dependent decrease of the characteristic absorbance of the ethanolic solution of DPPH at 518 nm in the presence of Qc and one of its derivatives, **Ia**, is illustrated by Figure 4. As shown in Figure 4, the initial fast absorbance decrease, corresponding to the transfer of the most labile H atoms, is followed by a much slower absorbance decline representing the residual antiradical activity of the antioxidant degradation products. The initial velocity of DPPH decolorization determined for the first 75-s interval was used as a marker of antiradical activity. Based on the kinetic parameter the compounds studied were arranged according to their decreasing activity in comparison with the parent Qc and standard trolox, as shown in Table 1. It is apparent that a group of six new derivatives (**Ia–Ie, IIa**) exert antioxidant activity comparable with that of Qc and even slightly higher. The antiradical efficacy of the most efficient chloronaphthoquinone derivative **Ia** was found comparable with that of the standard trolox. The results indicate that application of the initial velocity of DPPH decolorization allows good discrimination between the polyphenolic compounds studied. In addition, the kinetic parameter is considered to be of primary importance in antioxidant evaluation since fast reaction with low concentrations of short-living damaging radicals is of utmost importance for antioxidant protection. Other authors applied the kinetic approach to rank flavonoids according to their antioxidant efficacy (Goupy *et al.* 2003; Butkovic *et al.* 2004; Villano *et al.* 2007).

**Table 1:** Antiradical activities of novel quercetin derivatives, in comparison with parent quercetin and trolox standard, in a DPPH test^a^.

Compound	MW	Absorbance decrease (-ΔA/75 s)
*Ia* Chloronaphthoquinone Qc	492.82	0.462 ± 0.015
*Ib* Monochloropivaloyl Qc	420.80	0.446 ± 0.024
*Ic* Ditetraacquinoyl Qc	986.85	0.414 ± 0.021
*Id* Acetyldidiacetcaffeoyl Qc	836.72	0.391 ± 0.028
Quercetin	302.24	0.386 ± 0.025
*IIa* Diquercetin	602.46	0.374 ± 0.030
*Ie* Di(prenylferuloyl)Qc	790.82	0.316 ± 0.017
*If* Tetratrimetylgaloxyferuloyl Qc	1783.68	0.195 ± 0.011
*Ig* Triacetylcaffeoyl Qc	1040.9	0.121 ± 0.012
*Ih* Trichlorpivaloyl Qc	657.92	0.119 ± 0.030
*Ich* Didiizoprenocaffeoyl Qc	899.00	0.115 ± 0.031
*Ii* Tri-acetylferuloyl Qc	956.85	0.091 ± 0.012
*Ij* Acetylchlorogenoyl Qc	1437.25	0.063 ± 0.005
*Ik* Triacetylcaffeoyl Qc	998.86	0.043 ± 0.007
*Il* Monoacetylferuloyl Qc	520.45	0.040 ± 0.026
*Im* 3′-Morfolinohydroxypropoxy Qc	743.84	0.035 ± 0.021
*IIb* Heptabutyroyl biQc	1093.08	0.033 ± 0.012
*In* 5-Morfolinohydroxypropoxy Qc	473.47	0.014 ± 0.009
*Io* Tetra acetylsalicyloyl Qc	950.8	0.013 ± 0.003
*Ip* Pentaacetyl Qc	512.42	0.010 ± 0.009
*Ir* Trimethylgaloyl Qc	884.79	0.008 ± 0.004
*IIc* Hepta/hexaacetyldi Qc 1:1	896.71/854.68	0.007 ± 0.006
Trolox	-	0.520 ± 0.025

a The ethanolic solution of DPPH radical (50 µM) was incubated in the presence of the compound tested (50 µM). Absorbance decrease at 518 nm during the first 75-s interval was determined. Results are mean values ± SD from at least three measurements.

**Figure 5:**
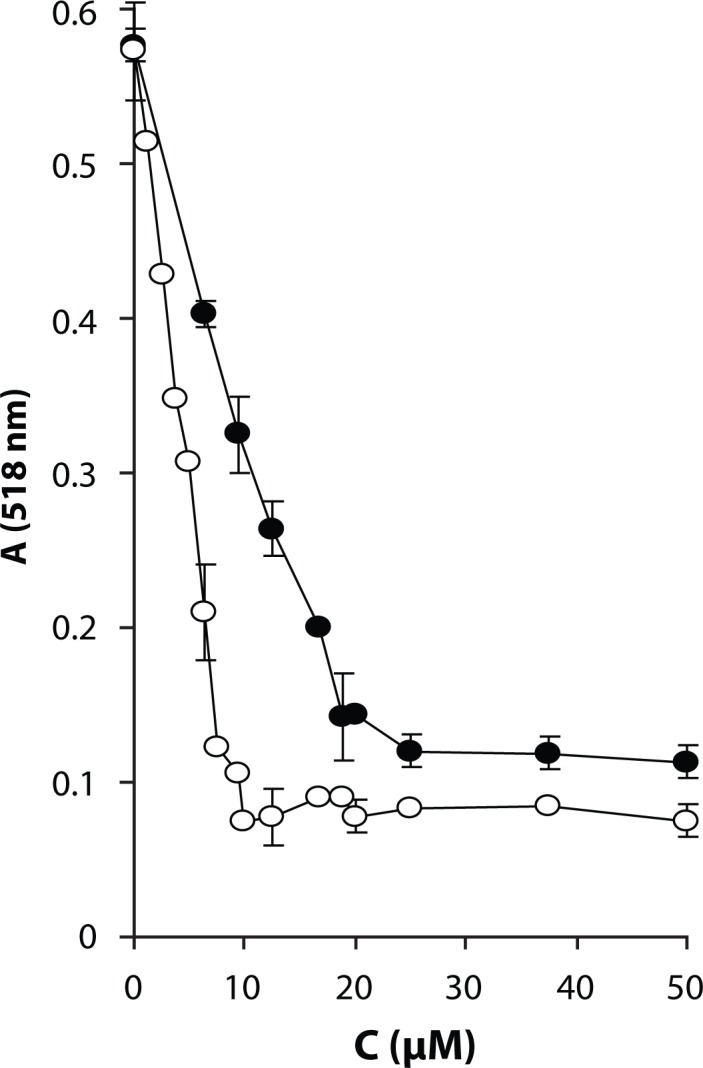
Stoichiometry of DPPH scavenging by the chloronaphthoquinone derivative of Qc **Ia** in comparison with Qc. Concentration dependence of absorbance decrease of ethanolic solution of DPPH radical (50 µmol/l) in the presence of increasing concentrations of the compounds tested at λ_max_ = 518 nm. (○) – Qc, time of reaction 1 h (●) – **Ia**, time of reaction 1.5 h. Results are mean values from two measurements or mean values±SD from three experiments.

In general, the antioxidant efficacy is characterized not only by kinetics of free radical quenching but also by stoichiometry of the scavenging reaction. So for the most efficient chloronaphthoquinone derivative **Ia**, the total stoichiometry of DPPH scavenging was determined in comparison with the parent Qc. The technique of spectrophotometric titration of fixed concentration of DPPH (50 µmol/l) with increasing concentrations of the antioxidant was used to determine the point of equivalence. In this approach the reaction time was set long enough to let the reaction run to completion. Fig. 5 shows the absorbance decrease of the ethanolic solution of DPPH radical in the presence of increasing concentrations of the compounds tested. By analyzing the titration curves, points of equivalence were determined and corresponding stoichiometric factors were calculated. Under the experimental conditions used, one molecule of **Ia** was found to quench 2.6±0.1 DPPH radicals, while one molecule of Qc scavenged 5.5±0.2 DPPH radicals. The high stoichiometric ratio found for Qc is in agreement with findings of other authors (Goupy *et al.* 2003; Villano *et al.* 2007; Markovic *et al.* 2012) and indicates high antiradical activity of its decomposition products which is in contrast to compound **Ia.** To conclude, by using a DPPH assay, 21 novel derivatives of Qc were ranked according to their antiradical efficacy in comparison with the parent Qc and the standard trolox. For the most efficient derivative, stoichiometry of DPPH scavenging was determined.
